# Increasingly inbred and fragmented populations of *Plasmodium vivax* associated with the eastward decline in malaria transmission across the Southwest Pacific

**DOI:** 10.1371/journal.pntd.0006146

**Published:** 2018-01-26

**Authors:** Andreea Waltmann, Cristian Koepfli, Natacha Tessier, Stephan Karl, Abebe Fola, Andrew W. Darcy, Lyndes Wini, G. L. Abby Harrison, Céline Barnadas, Charlie Jennison, Harin Karunajeewa, Sarah Boyd, Maxine Whittaker, James Kazura, Melanie Bahlo, Ivo Mueller, Alyssa E. Barry

**Affiliations:** 1 Division of Population Health and Immunity, The Walter & Eliza Hall Institute of Medical Research, Melbourne, Australia; 2 Department of Medical Biology, University of Melbourne, Melbourne, Australia; 3 The National Health Training and Research Institute, Ministry of Health, Honiara, Solomon Islands; 4 National Vector Borne Disease Control Program, Ministry of Health, Honiara, Solomon Islands; 5 Division of Tropical Health and Medicine, James Cook University, Townsville, Australia; 6 Center for Global Health and Diseases, Case Western Reserve University, Cleveland, Ohio, United States of America; 7 Parasites and Insect Vectors Department, Institut Pasteur, Paris, France; University of Sao Paolo, BRAZIL

## Abstract

The human malaria parasite *Plasmodium vivax* is more resistant to malaria control strategies than *Plasmodium falciparum*, and maintains high genetic diversity even when transmission is low. To investigate whether declining *P*. *vivax* transmission leads to increasing population structure that would facilitate elimination, we genotyped samples from across the Southwest Pacific region, which experiences an eastward decline in malaria transmission, as well as samples from two time points at one site (Tetere, Solomon Islands) during intensified malaria control. Analysis of 887 *P*. *vivax* microsatellite haplotypes from hyperendemic Papua New Guinea (PNG, n = 443), meso-hyperendemic Solomon Islands (n = 420), and hypoendemic Vanuatu (n = 24) revealed increasing population structure and multilocus linkage disequilibrium yet a modest decline in diversity as transmission decreases over space and time. In Solomon Islands, which has had sustained control efforts for 20 years, and Vanuatu, which has experienced sustained low transmission for many years, significant population structure was observed at different spatial scales. We conclude that control efforts will eventually impact *P*. *vivax* population structure and with sustained pressure, populations may eventually fragment into a limited number of clustered foci that could be targeted for elimination.

## Author summary

*Plasmodium vivax* is a major human malaria parasite, common in endemic areas outside sub-Saharan Africa, and more difficult to control than other malaria parasite species. The distinct lifecycle biology of *P*. *vivax* is thought to contribute to its more stable and efficient transmission allowing the maintenance of high diversity and potentially, gene flow. Independent studies are therefore needed to understand how *P*. *vivax* populations respond to changing transmission levels, in order to inform malaria control and elimination efforts. Here we have determined parasite population genetic structure in three countries of the Southwest Pacific, an island chain with a natural west to east decline in transmission intensity (Papua New Guinea > Solomon Islands > Vanuatu). With declining transmission, *P*. *vivax* populations experience only a modest decline in diversity but a significant increase in multilocus linkage disequilibrium and population structure, indicating that parasite populations become more inbred and begin to fragment into clustered foci. Analysis of two time points in one study area (Tetere, Solomon Islands) also show similar changes in association with intensifying malaria control. The results indicate that with long term sustained malaria control *P*. *vivax* populations will eventually fracture into population clusters that could be targeted for elimination.

## Introduction

The international intensification of malaria control over the last 15 years has reduced the global malaria burden by more than 50% with rapidly declining transmission in many endemic regions [1]. *Plasmodium falciparum* and *Plasmodium vivax* are the major agents of human malaria however *P*. *vivax* is becoming the main source of malaria infection and disease in co-endemic areas because it is more resilient to control efforts [1–9]. These shifts in species dominance may result from the fact that *P*. *vivax* employs unique transmission strategies including dormant liver-stage infections that relapse months to years after the primary infection [10]. These biological characteristics suggest that *P*. *vivax* will be the far more challenging species to eliminate [10–13], and that interventions and monitoring approaches originally developed for *P*. *falciparum* malaria may not be sufficient or suitable for *P*. *vivax* [6, 14–17].

Surveillance tools that monitor the impact of antimalarial interventions are central to determining the success of disease control programs. Population genetics has been successfully harnessed to understand local changes in *P*. *falciparum* transmission dynamics in response to sustained control [18], but this has not yet been applied extensively to *P*. *vivax*. Plasmodium parasites are haploid in the human host and replicate asexually for most of the lifecycle but undergo sexual replication and a brief period of diploidy within the mosquito vector. During this stage, meiosis produces haploid recombinant progeny that are then inoculated back into the human host. The co-transmission of multiple genetically distinct clones to the vector is thus central to the generation and maintenance of diversity via sexual recombination [19, 20]. As infections decline both within and among hosts, it is expected that effective population size, genetic diversity and gene flow will decrease, eventually leading to inbred, structured populations [21–23]. Conversely, in areas of high transmission, recombination between distinct clones and gene flow are more common, resulting in diverse, unstructured populations [22]. Whilst *P*. *falciparum* fits this expectation [22], *P*. *vivax* populations retain high levels of diversity and large effective population sizes at low transmission [7, 24–28] and have higher diversity than *P*. *falciparum* populations [5, 29–31]. *P*. *vivax* population structure has been reported for some areas [27, 32–34], but is absent in others [5, 30, 35, 36] and does not appear to be associated with the level of transmission. The population structure observed in countries such as Peru [27], Colombia [32] and Malaysia [33], can be explained by multiple independent introductions of the parasite [37], historically low *P*. *vivax* transmission [27, 34], non-overlapping vector species refractory to non-autochthonous *P*. *vivax* strains [38] and historically focal transmission combined with recent reductions due to control [33]. In regions with past hyperendemic *P*. *vivax* transmission and recent upscaling of malaria control efforts, population structure has not been observed [5]. The relationship between *P*. *vivax* transmission and population genetic parameters thus remains poorly understood, and requires systematic investigations with declining transmission and in the context of long-term intensified control.

Historically, the Southwest Pacific region, in particular Papua New Guinea (PNG) and Solomon Islands, has endured some of the highest *P*. *vivax* transmission anywhere in the world [39, 40]. This region has a natural, gradual decline in malaria endemicity from west to east with high transmission in PNG, moderate-to-high in Solomon Islands and low transmission in Vanuatu [39], that has been accentuated by recent control efforts [7, 41, 42]. Our previously published population genetic data from PNG and Solomon Islands [30, 35], combined with new samples from ongoing studies within Solomon Islands and Vanuatu, presents a unique opportunity to understand the population genetics of *P*. *vivax* in context with declining transmission. Here we have defined *P*. *vivax* population genetic structure at different transmission intensities, spatial scales and in the context of successful long-term malaria control. We analysed almost 900 *P*. *vivax* microsatellite haplotypes from *P*. *vivax* isolates collected from infected humans throughout the Southwest Pacific region, including dense spatial and temporal sampling in the Solomon Islands [7]. The results demonstrate that *P*. *vivax* exhibits significant changes in population genetic parameters with declining transmission over space and time, highlighting the importance of maintaining control efforts, and the key role that population genetic surveillance of *P*. *vivax* can play in malaria control and elimination.

## Materials and methods

### Study area and samples

**Fig 1:**
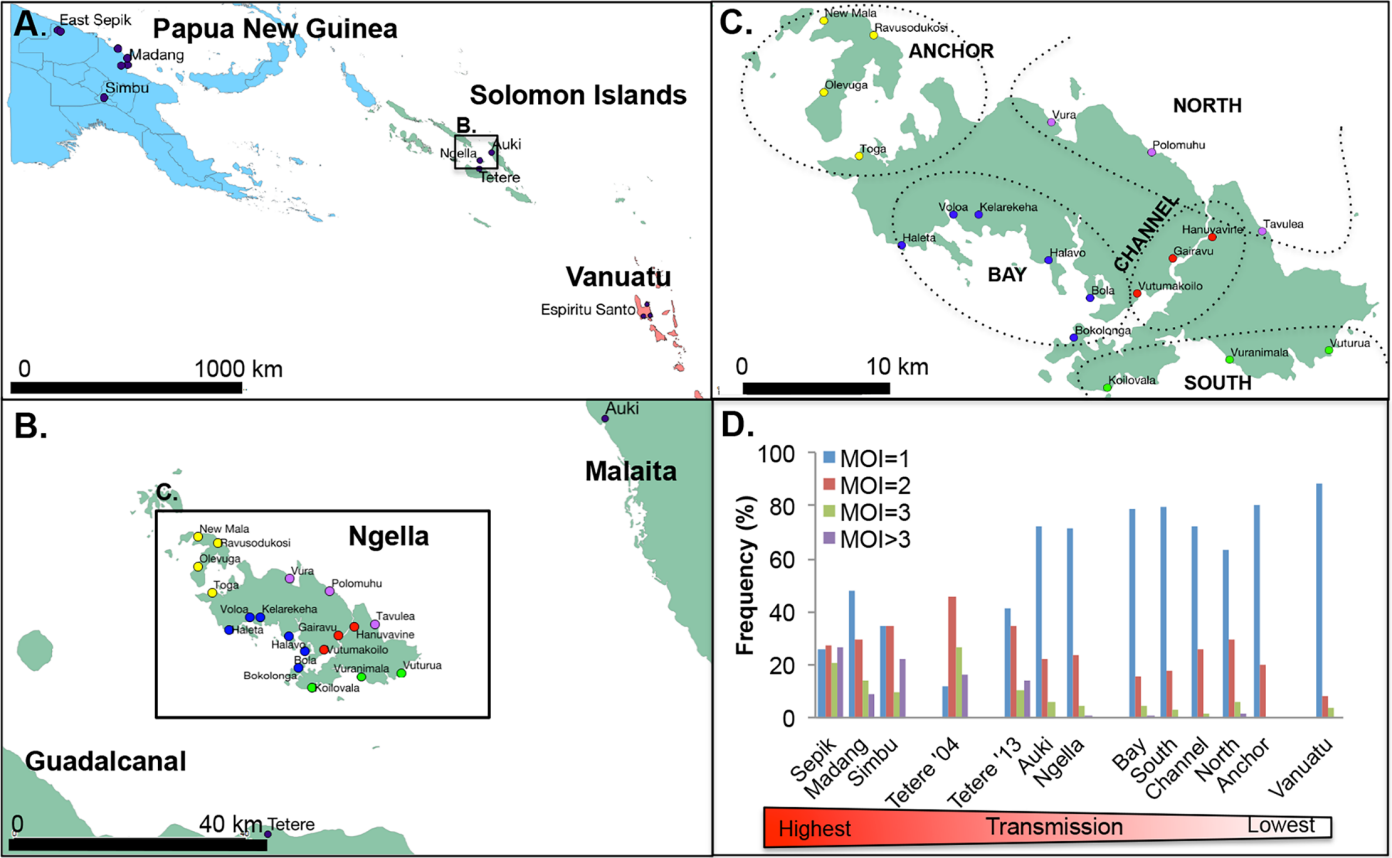
Map of the study areas and transmission intensity. (A) Southwest Pacific sampling locations showing Papua New Guinea in blue, Solomon Islands in green and Vanuatu in red, (B) central Solomon Islands, (C) Ngella, showing 19 villages and five distinct geographical/ecological regions. Anchor villages are indicated in yellow, Bay in blue, South Coast in green, Channel in red and North Coast in purple). Maps were produced using an open source map with shape files downloaded from diva-gis.org using the open source software QGIS release 2.18. (D) The distribution of multiplicity of infection values in each defined population, shown as an indicator of *P*. *vivax* transmission intensity.

*P*. *vivax* positive samples from PNG, Solomon Islands and Vanuatu were used in this study (Fig 1). PNG has traditionally had the highest burden of the three countries and control has only been intensified in the last ten years through universal access to long lasting insecticide treated bednets (LLIN) and access to artemisinin combination therapy (ACT) [43]. In Solomon Islands, sustained and intensified malaria interventions in the last two decades including LLIN, indoor residual spraying and ACT have resulted in an approximately 90% reduction in malaria incidence [1, 2, 7, 44]. The small country of Vanuatu harbors the southern boundary of malaria transmission in the Pacific, as it is crossed by the Buxton Line, which defines the limit of Anopheline breeding [45] resulting in a very low clinical infection rate that is dominated by *P*. *vivax* infections [1]. At the time of sampling, transmission ranged from high in PNG (prevalence = 17.0–31.7%), moderate-high in Solomon Islands (3.9–31.7%) and low in Vanuatu (<1% [39], S1 Table).

Genotyping data from total of 887 *P*. *vivax* isolates from PNG (n = 443), Solomon Islands (n = 420) and Vanuatu (n = 24) were obtained (Table 1, S2 File). *P*. *vivax* positive samples were from both clinical and asymptomatic infections collected during different epidemiological surveys (S1 Table). Data included previously published genotyping data from PNG collected in 2005–6 (n = 443) and Solomon Islands in 2004–5 (Tetere 2004, n = 45) [30, 35] in addition to 375 newly typed *P*. *vivax* isolates from three provinces of the Solomon Islands collected in 2012–2013 [7], and 24 genotypes from one province of Vanuatu collected in 2013 (Fig 1A). Dense sampling of the central region of the Solomon Islands allowed analyses at different spatial scales in three neighbouring island provinces including Guadalcanal (Tetere 2013, n = 39), Malaita (Auki, n = 13) and Central Province (Ngella, n = 323) (Fig 1B). In Ngella, sampling included 19 villages organized into five geographically and ecologically distinct areas including Bay (n = 83), South (n = 35), Channel (n = 46), North (n = 136) and Anchor (n = 23, Fig 1C). In Vanuatu, samples were collected from the province of Espiritu Santo and included the villages of Port Orly (n = 7), Luganville (n = 7) and Nambauk (n = 10). Further details of the samples and study sites are summarised in S1 Table and S1 File. All data and samples were de-identified for the analysis.

**Table 1:** Genetic diversity of *Plasmodium vivax* populations of the Southwest Pacific.

Country	Province	Population	*n*	*A*±SEM	*H*_s_±SEM	*R*_S_±SEM	*P*_*S*_ >0.50	*N*_e_ (95% CI)	*N*_e_ (95% CI)
								SMM	IAM
Papua New Guinea	East Sepik		229	13.44±0.38	0.81±0.006	8.75±0.20	0.013	16059 (6901, 36582)	6811 (2927, 15515)
	Madang		175	15±0.39	0.84±0.005	9.62±0.20	0.015	24293 (10439, 55337)	8234 (3539, 18757)
	Simbu		39	8±0.46	0.81±0.011	7.37±0.38	0.015	15539 (6678, 35397)	6713 (2885, 15291)
Solomon Islands	Guadalcanal	Tetere 2004	45	9.44±0.53	0.84±0.009	8.44±0.43	0.016	23220 (9978, 52893)	8061 (3464, 18362)
		Tetere 2013	39	7.78±0.3	0.79±0.009	7.05±0.24	0.034	11766 (5056, 26802)	5963 (2562, 13583)
	Central Islands (Ngella)	Bay	83	12.33±0.27	0.81±0.006	9.20±0.15	0.015	16112 (6924, 36703)	6821 (2931, 15538)
		South	35	9.11±0.34	0.82±0.012	8.50±0.32	0.015	16599 (7133, 37811)	6911 (2970, 15744)
		Channel	46	9.33±0.35	0.79±0.009	8.10±0.28	0.012	11500 (4942, 26196)	5907 (2538, 13456)
		North	136	13.89±0.28	0.85±0.004	9.73±0.17	0.013	26387 (11339, 60107)	8564 (3680, 19509)
		Anchor	23	6.56±0.38	0.79±0.019	6.51±0.37	0.011	10771 (4629, 24535)	5752 (2472, 13103)
	Malaita	Auki	13	5.33±0.54	0.80±0.026	n.a.	0.097	13170 (5659, 30000)	6250 (2686, 14238)
Vanuatu	Sanma	Espiritu Santo	24	5.56±0.34	0.72±0.022	5.45±0.33	0.034	4056 (1743, 9239)	4131 (1775, 9411)
TOTAL			887	n.d.	n.d.	n.d.	n.d.	n.d.	n.d.

*n* = number of microsatellite genotypes, *A* = number of alleles, *H*_*s*_ = gene diversity, *R*_s_ = allelic richness based on smallest sample size, *P*_S_ >0.50 = proportion of pairs with relatedness greater than 0.50, *N*_e_ = effective population size, SEM = standard error of the mean, SMM = Stepwise mutation model, IAM = Infinite alleles mode, n.a. = excluded due to small sample size, n.d. = not done.

### Ethics statement

The study was approved by The Walter and Eliza Hall Institute Human Research Ethics Committee (12/01, 11/12 and 13/02), the Papua New Guinea Institute of Medical Research Institutional Review Board (11–05), the Papua New Guinea Medical Research Advisory Committee (11–06), the Solomon Islands National Health Research Ethics Committee (12/022) and the Vanuatu Ministry of Health (19-02-2013).

### Multiplicity of Infection (MOI)

To allow the selection of low complexity infections for confident reconstruction of haplotypes, we first determined the multiplicity of infection (MOI) in each *P*. *vivax* isolate by genotyping with the highly polymorphic microsatellites, MS16 and *msp1*F3 [30, 35]. MOI data was previously published for the PNG [30, 35], Tetere 2004 [35], and Ngella datasets [7]. The MOI in the Tetere 2013, Auki 2013, and Vanuatu *P*. *vivax* populations was determined for this study, and done according to previously published protocols [30, 35]. Sample numbers that were genotyped using this approach are indicated in S1 Table.

### Multilocus microsatellite genotyping

To measure population structure, all confirmed monoclonal infections (MOI = 1) from new Solomon Islands (Tetere 2013, Ngella and Auki) and Vanuatu samples were genotyped with nine genome-wide and putatively neutral microsatellites loci (MS1, MS2, MS5, MS6, MS7, MS9, MS10, MS12 and MS15) [46]. Due to small sample size, Auki and Vanuatu populations were supplemented by genotyping additional low complexity polyclonal infections (MOI = 2). Previously published data on the nine microsatellite markers for PNG and Tetere 2004 isolates was also derived from low complexity samples (MOI = 1 or 2, [30, 35]). A semi-nested PCR strategy was employed, whereby a multiplex primary PCR was followed by nine individual secondary reactions, with a fluorescently labelled forward primer, as previously described [30, 35]. PCR products were sent to a commercial facility for GeneScan fragment analysis on an ABI3730xl capillary electrophoresis platform (Applied Biosystems) using the size standard LIZ500.

### Data analysis

Electropherograms resulting from the fragment analysis were visually inspected and the sizes of the fluorescently labeled PCR products were scored with Genemapper V4.0 software (Applied Biosystems), with the peak calling strategy done as previously described [30]. Raw data from the published dataset was added to the new dataset and binned together to obtain consistent allele calls. Automatic binning (i.e. rounding of fragment length to specific allele sizes) was performed with Tandem [47]. After binning, quality control for individual *P*. *vivax* haplotypes and microsatellite markers was conducted to confirm the markers were not in linkage disequilibrium (LD) and to identify outlier haplotypes and/or markers (i.e. haplotypes or markers which are disproportionately driving variance in the dataset). Isolates with one allele at all markers, or more than one allele at only one microsatellite marker were considered “confirmed monoclonal infections”. For isolates with more than one allele at any of the loci, the dominant alleles (highest peaks) were used to construct “dominant haplotypes” as previously described [30]. Both monoclonal infection and dominant haplotypes were combined for population genetic analyses [30, 35].

Allele frequencies and input files for the various population genetics software programs were created using CONVERT version 1.31. Allele frequencies and genetic diversity parameters including the number of alleles (*A*) and Nei’s unbiased estimator of gene diversity (*H*_s_) [48] were measured using *FSTAT* version 2.9.3.2 [49]. Because *A* is influenced by sample size we also calculated the allelic richness (*R*_s_), which is normalized on the basis of the smallest sample size and based on the rarefaction method developed by Hurlbert [50] as implemented in *FSTAT* version 2.9.3.2 [49]. In addition, we measured the pairwise relatedness between haplotypes (*P*_S_), calculated by determining the proportion of alleles shared between haplotypes as a function of the total number of markers genotyped. The proportion of pairs with *P*s values greater than 0.50 (*P*s>0.50), was then used as an indicator of relatedness within populations, and is analogous to Identity by Descent measures used by Taylor *et al*. and shown to decay with geographic distance [51]. Effective Population Size (N_e_) was calculated using the stepwise mutation model (SMM) and infinite alleles model (IAM), as previously described [22]. Mutation rates for *P*. *vivax* were not available and thus the *P*. *falciparum* mutation rate was used [52]. For SMM, N_e_ was calculated as follows: $N_{e}=\frac{\frac{1}{8}x\left\{{\left[\frac{1}{1-H_{E_{mean}}}\right]}^{2}-1\right\}}{\mu }$ where *H*_E mean_ is the expected heterozygosity averaged across all loci. For the IAM, *N*_e_ was calculated using the formula: $N_{e}=\left(\frac{H_{E_{mean}}}{4\left(1-H_{E_{mean}}\right)}\right)x\frac{1}{\mu }$. As a measure of inbreeding in the populations studied, multilocus LD (non-random associations between alleles of all pairs of markers) was estimated using the standardized index of association (*I*_A_^S^) in LIAN version 3.6. *I*_A_^S^ compares the observed variance in the number of shared alleles between parasites with that expected under equilibrium, when alleles at different loci are not in association [53]. The measure was followed by a formal test of the null hypothesis of LD and *p*-values were derived. Only unique haplotypes with complete genotypes were used and Monte Carlo tests with 100,000 re-samplings were applied [53]. The number of unique haplotypes was assessed using DROPOUT [54]. To confirm that LD was not artificially reduced by false reconstruction of dominant haplotypes, the analysis was performed for the combined dataset of dominant and monoclonal infection haplotypes (i.e. all haplotypes), and for monoclonal infection haplotypes only. MS2 and MS5 both localize to chromosome 6 and MS12 and MS15 to chromosome 5 thus, analyses were repeated on datasets where MS5 and MS15 were excluded (chosen due to a greater degree of missing data) using the remaining seven loci spanning seven chromosomes. Where sample size permitted (n > 5), multilocus LD was also estimated at the village level.

To investigate geographic population structure, for each metapopulation we measured the weighted average F-statistics over all loci using the distance method [55] using global AMOVA implemented in Arlequin version 3.5.2.2 [56]. Pairwise comparisons among populations were done using three measures of genetic differentiation, namely *F*_ST_, *G*_ST_ and Jost’s *D*. *F*_ST_ was estimated using FSTAT version 2.9.3.2 [49]. G_ST_ [57] and Jost’s *D* [58] were estimated using the *R* package *DEMEtics*, as previously described [59]. Population structure was further confirmed by Bayesian clustering of haplotypes implemented in the software STRUCTURE version 2.3.4 [60], and was used to investigate whether haplotypes cluster into distinct genetic populations (*K*) among the defined geographic areas. The analyses were run for *K* = 1–20, with 20 independent stochastic simulations for each *K* and 100,000 MCMCs, after an initial burn-in period of 10,000 MCMCs using the admixture model and correlated allele frequencies. The results were processed using STRUCTURE Harvester [61], to calculate the optimal number of clusters as indicated by a peak in Δ*K* according to the method of Evanno *et al*. [62]. The programs CLUMPP version 1.1.2 [63] and DISTRUCT 1.1 [64] were used to display the results. To assess phylogenetic clustering of haplotypes in each geographic area, the R software (APE) package was used to draw an unrooted phylogenetic tree using pairwise distances between multilocus haplotypes [65].

Statistical analysis of epidemiological and population genetic parameters was done using Graphpad Prism version 7.

## Results

### Wide range of *Plasmodium vivax* transmission intensities across the study area

Based on infection prevalence data, PNG, Solomon Islands and Vanuatu represent high, moderate to high, and low transmission areas respectively ([39], S1 Table, S1 File). Because reliable prevalence data was not available for all populations, as an additional measure of transmission intensity we determined the multiplicity of infection (MOI) and examined the frequency distribution of samples with 1, 2, 3 or >3 clones, and the proportions of polyclonal infections in each population. MOI was determined by genotyping of all available *P*. *vivax* infections using the highly polymorphic markers *MS16* and *msp1*F3, and the proportion of polyclonal infections in each population [66](S1 Table). The MOI frequency distribution varied significantly across the Southwest Pacific (Fig 1D, Chi Squared test: p<0.0001) with polyclonal infections ranging from high in PNG (52.2%-74.3%), and moderate to high in Solomon Islands (28.6–88.2%) to low in Vanuatu (12%, S1 Table). The Solomon Islands population of Tetere experienced a significant change in the frequency distribution over a period of intensive control with polyclonal infections declining between 2004 (88.2%) to 2013 (58.6%, Fig 1D, S1 Table, Chi Squared test: p = 0.0014). There was significant variability in the distribution of polyclonal infections also among subpopulations of both PNG and Solomon Islands (Chi Squared test: p<0.0001). In the Solomon Islands, Tetere 2013 (58.6%) had a higher proportion of polyclonal infections than Auki (28.6%) and Ngella (30.0%), consistent with lower transmission in the latter two regions. Within Ngella, an area of dense sampling divided into five distinct ecological zones (Anchor, North, Channel, South and Bay), the proportion of polyclonal infections ranged between 20.0–36.9% (S1 Table), and was significantly associated with prevalence (Linear regression: r^2^ = 0.97, p = 0.002).

### Definition of microsatellite haplotypes

Low complexity isolates (MOI = 1 or 2) were selected for further characterization with the full panel of nine-microsatellites. This strategy increases confidence in multilocus haplotypes, and since the majority of infections are MOI = 1 it was possible to reconstruct haplotypes from large numbers of samples with high confidence. New haplotypes were obtained for all monoclonal infections (MOI = 1) from the Solomon Islands Tetere 2013, Ngella and Auki populations, and from Vanuatu by genotyping an overlapping set of nine microsatellite markers. Two low complexity polyclonal infections (MOI = 2) each from Auki and Vanuatu were also genotyped to boost sample numbers in those populations. Published microsatellite haplotype data was available for PNG (n = 443) and the Tetere 2004 population (n = 45, [30, 35]). Only high-quality haplotypes with data for at least five out of nine microsatellite loci were retained for population genetic analysis [30, 35], resulting in seven haplotypes being excluded. Two further haplotypes were identified as outliers (i.e. those that do not conform to the expected distribution) due to rare singleton alleles at the MS2 locus, and were discarded for subsequent analyses. The final dataset comprised a total of 887 haplotypes including 443 from PNG, 420 from Solomon Islands and 24 from Vanuatu (Table 1, S1 Table). The microsatellite haplotype dataset is available as a supporting file (S2 File) for further analyses however caution is needed if comparing to other datasets, since allele calls need to be binned together using raw data. Although most samples were initially identified as monoclonal, multiple alleles were detected after genotyping the additional nine markers. Therefore, the data includes 555 confirmed monoclonal infection haplotypes and 332 “dominant” haplotypes comprising the dominant allele calls (highest peaks) from samples with multiple alleles. The 887 haplotypes were distributed across all catchment areas, as were the 332 dominant haplotypes, however small sample sizes were available for lower prevalence regions of Auki and Vanuatu (Table 1). Note that *MS16* and *msp1F3* were used only to determine MOI and are not recommended for analysis of population structure due to their extreme diversity [67, 68] and therefore they were excluded for the following analyses.

### Diversity and effective population size

**Fig 2:**
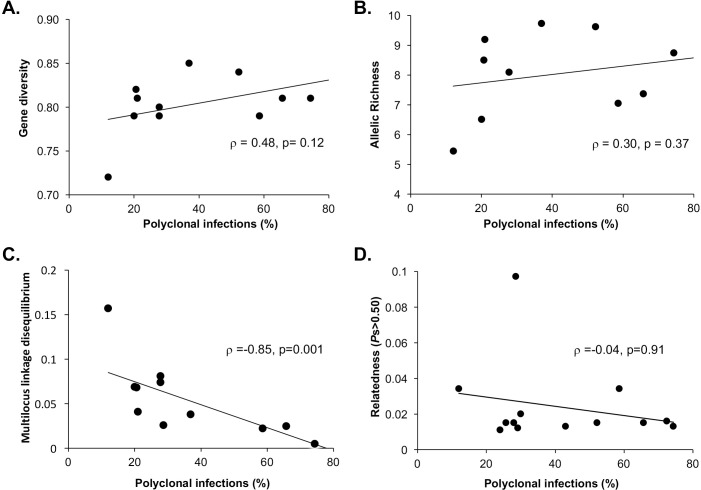
Relationship between transmission intensity and population genetic parameters for *Plasmodium vivax* populations of the Southwest Pacific. Diversity of parasite populations based on (A) mean gene diversity (*H*_s_), (B) Allelic richness (*R*_s_), (C) the proportion of closely related haplotype pairs (*P*s>0.50) and (D) multilocus linkage disequilibrium (*I*_A_^S^) was plotted against the proportion of polyclonal infections for each defined population (see Table 1 and S1 Table).

Mean genetic diversity of the microsatellite markers showed a modest but significant trend of declining diversity from PNG (*H*_S_ = 0.81–0.84, *R*_S_ = 7.37–9.62) to Solomon Islands (*H*_S_ = 0.79–0.85, *R*_S_ = 6.51–9.20) and Vanuatu (*H*_S_ = 0.72, *R*_S_ = 5.45) (One way ANOVA test of trend: p <0.05, Table 1). There was a trend of decreasing population diversity (*H*_S_, *R*_S_) and increasing proportions of closely related haplotypes (*P*s>0.50) with declining polyclonal infections but this was not significant (Fig 2A–2C). In addition, effective population sizes (*N*_*e*_) reflect the high diversity across the different parasite populations. The Solomon Islands and PNG populations showed moderate to high *N*_*e*_, while Vanuatu had 1.5–5 fold lower *N*_e_ than any of the other populations (Table 1). The patterns observed suggest that sustained low transmission, such as that seen in Vanuatu, is needed for significant reductions in diversity and effective population size.

### Multilocus linkage disequilibrium

Multilocus linkage disequilibrium of asexual blood stage parasites is an indirect measure of the rate of recombination between related individuals (inbreeding) in the mosquito stages, which is expected as transmission declines and infections become increasingly clustered. In previously published data from the high transmission sites of PNG and the earlier Solomon Islands timepoint (Tetere 2004) there were no identical haplotypes and no significant multilocus LD was observed indicating limited inbreeding and random associations between alleles in those populations [30, 35]. In the later data from Solomon Islands (i.e. Tetere 2013, Ngella and Auki) and Vanuatu, seven haplotypes were found repeatedly amongst 22 isolates, suggesting clonal transmission due to self-fertilization and no detectable recombination, or alternatively, a single mosquito infecting several individuals. All repeated haplotypes were found in Ngella, and four were distributed among different villages or regions (S1 Fig), making the latter scenario unlikely. Repeated and incomplete haplotypes were excluded for the analysis of multilocus LD retaining only the unique, complete microsatellite haplotypes comprised of all nine markers (n = 248). Significant multilocus LD was observed in the contemporary Solomon Islands populations (Tetere 2013, Ngella and Auki), including the five Ngella subpopulations, and in Vanuatu (Table 2) [30, 35]. The pattern of multilocus LD was retained when only monoclonal haplotypes from the dataset were considered (n = 93, Table 2), as well as when only one locus per chromosome was analyzed, confirming that LD was not the result of false reconstruction or physical linkage, respectively (S2 Table). Multilocus LD was significantly inversely associated with the proportion of polyclonal infections (Fig 2D). Thus, multilocus LD is present only in the post-control Tetere 2013 population and in low transmission populations of Auki, Ngella and Vanuatu. This suggests increasing LD with declining transmission due to geospatial variability, and malaria intervention in Tetere.

**Table 2:** Estimates of Multilocus Linkage Disequilibrium (LD) in *Plasmodium vivax* populations of the Southwest Pacific.

Population	Subpopulation	All haplotypes, all loci			Confirmed monoclonal haplotypes, all loci		
		*n*	*I*_A_^S^	*p*	*n*	*I*_A_^S^	*p*
**Tetere 2004**		21	0.012	0.18	0	n.a.	n.d.
**Tetere 2013**		31	0.022	0.0042	16	0.033	0.0183
**Auki**		9	0.081	0.005	6	0.054	0.13
**Ngella**		165	0.026	<0.00001	61	0.043	<0.00001
	Bay	32	0.041	0.0008	9	0.092	0.0052
	South	17	0.068	0.0003	7	0.163	0.0005
	Channel	29	0.074	<0.00001	9	0.087	0.0053
	North	73	0.038	<0.00001	28	0.076	<0.00001
	Anchor	14	0.069	0.0009	8	0.046	0.0948
**Vanuatu**		22	0.157	<0.00001	10	0.169	<0.00001
**TOTAL**		248	n.d.	n.d.	93	n.d.	n.d.

Multilocus LD values from PNG and Tetere 2004 populations are published elsewhere [35]. Complete and unique haplotypes only were used for the analysis, by discarding all but one of the seven clonal haplotypes identified. *n* = number of haplotypes used in the analysis. *I*_A_^S^ = Index of Association from LIAN analysis, MOI = multiplicity of infection, n.a. = not available due to sample size constraints, n.d. = not done.

### Population structure

**Fig 3:**
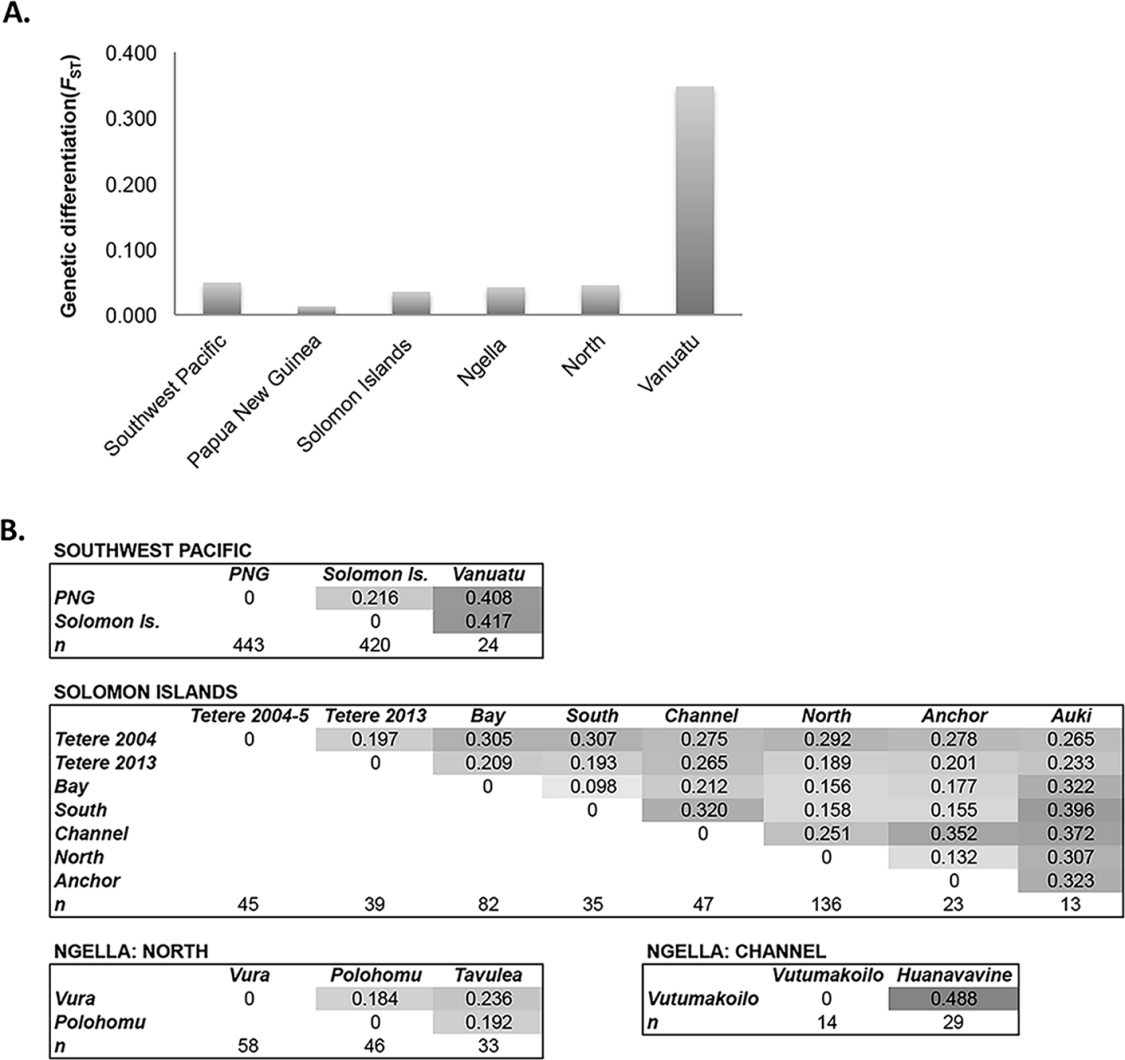
Genetic differentiation of *Plasmodium vivax* populations across the Southwest Pacific. (A) Average genetic differentiation among subpopulations. Average F statistics (*F*_ST_) was measured over all loci for all regions with at least two sub-populations of 20 or more samples, with the exception of Vanuatu, which had three populations of 7–10 samples. (B) Pairwise genetic differentiation between subpopulations. Pairwise differentiation was measured using Jost’s D, which accounts for the high diversity of microsatellite markers [58]. Values are shown for populations at different spatial scales. Darker shading indicates higher values.

To measure population structure across the study area, patterns of genetic differentiation among populations and clustering of haplotypes was investigated. Average F-statistics over all loci indicated the presence of low levels of population subdivision amongst countries (*F*_ST_ = 0.049) and a gradient of increasing structure from high to low transmission. Negligible differentiation was observed among provinces in PNG (East Sepik, Madang and Simbu: *F*_ST_ = 0.013), low levels among Solomon Islands provinces (Tetere 2013, Auki, Ngella: *F*_ST_ = 0.035), and slightly higher genetic differentiation was observed among Ngella regions (Bay, South, Channel, North, Anchor: *F*_ST_ = 0.042, Fig 3A). Very high genetic differentiation was found among the three Vanuatu villages (Port Orly, Nambauk, Luganville: *F*_ST_ = 0.348, Fig 3A), however sample sizes were much smaller for this country (n per village = 7–10), making this analysis less reliable, with potentially inflated *F*_ST_. Despite dense sampling within the Ngella regions, sample sizes were too small for village-level analysis within all but the North Coast region where it was similar to that found among the five Ngella zones (*F*_ST_ = 0.045, Fig 3A). Pairwise Jost’s *D* statistics, which account for the high diversity of microsatellites [30, 58]), confirm moderate to high differentiation among countries with 22–42% private alleles (Fig 3B). Within Solomon Islands, moderate to high proportions of private alleles were observed for Ngella: Channel (21–31%) and Auki (27–40%) compared to other populations. In addition, there was moderate genetic differentiation between villages on the Ngella:North Coast (18–24%) and high differentiation between Channel villages (49%)(Fig 3B). Pairwise *G*_ST_ and *F*_ST_ values are also provided in the Supporting Information for comparison to other studies (S3 Table).

**Fig 4:**
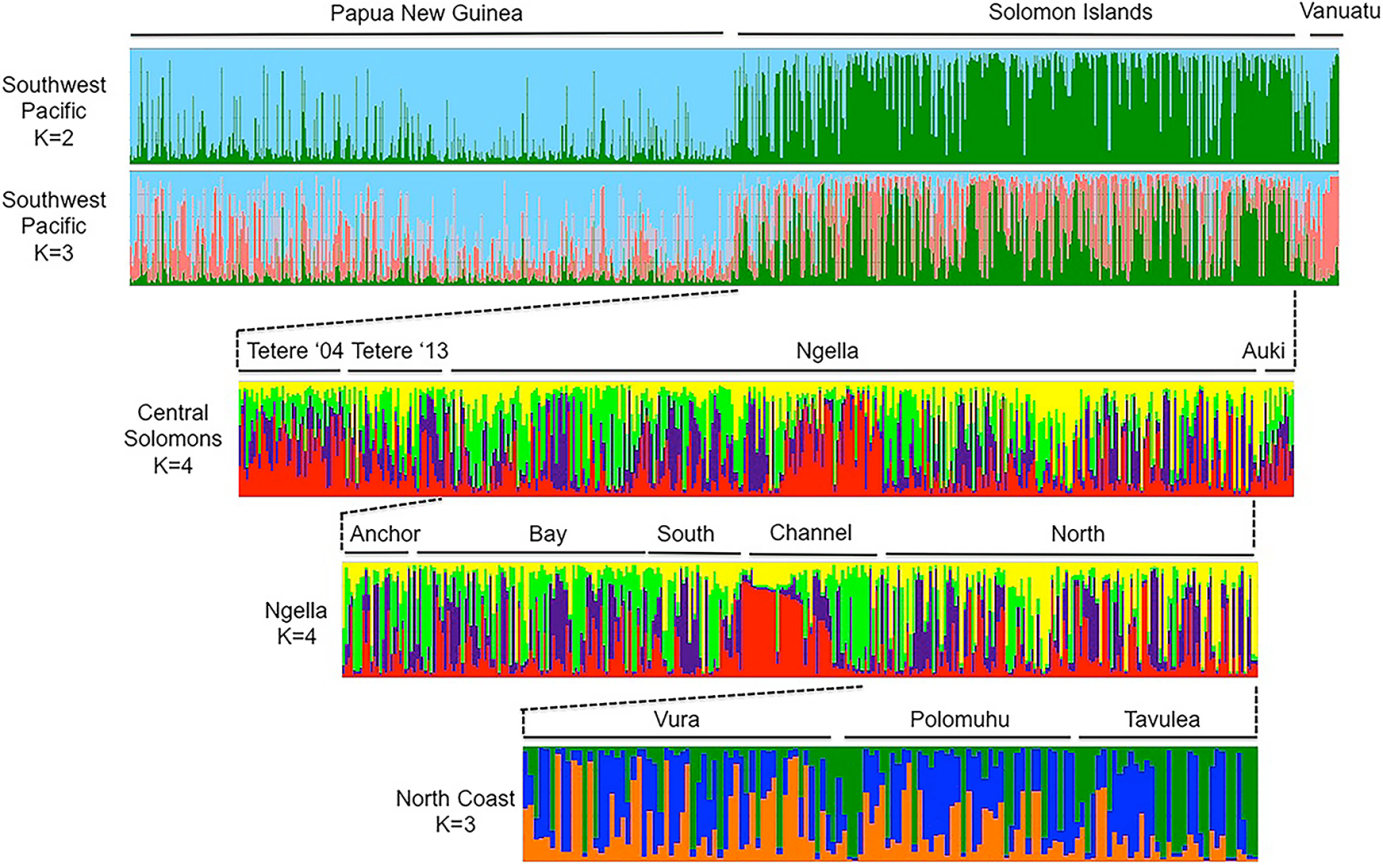
Geographical population clustering of *Plasmodium vivax* isolates of the Southwest Pacific. Results of STRUCTURE analysis are shown for different geographic strata. The analysis assigns *P*. *vivax* haplotypes to a defined number of genetic clusters (*K*) based on genetic distance. Vertical bars indicate individual *P*. *vivax* haplotype and colours represent the ancestry co-efficient (membership) within each cluster.

To investigate haplotype clustering patterns, we used the program STRUCTURE to define up to 20 genetic clusters (K = 1–20) within the entire dataset, as well as for Solomon Islands and its sub-regions. The analysis identified a small number of sub-populations at various spatial scales down to the village level (Fig 4, S2 Fig). A major subdivision in Southwest Pacific parasites occurs at K = 2 between PNG and Solomon Islands and is supported by the ΔK analysis, whilst Vanuatu appears to be a mixture of the two (Fig 4, S2 Fig). A ΔK peak at K = 2 can be an artifact of STRUCTURE analyses especially where strong population structure occurs at the highest hierarchy. At K = 3 however, samples from the three countries cluster into three genetically distinct populations (K = 3) (Fig 4, S2 Fig). For Solomon Islands, further substructure was observed at K = 4, and within Ngella: Channel, with the Hanuvavine and Vutumakoilo village haplotypes forming distinct clusters; and on the North Coast, with some genetic clustering observed amongst villages (Fig 4, S2 Fig). Vanuatu haplotypes appear to cluster into two major groups also (Fig 4).

**Fig 5:**
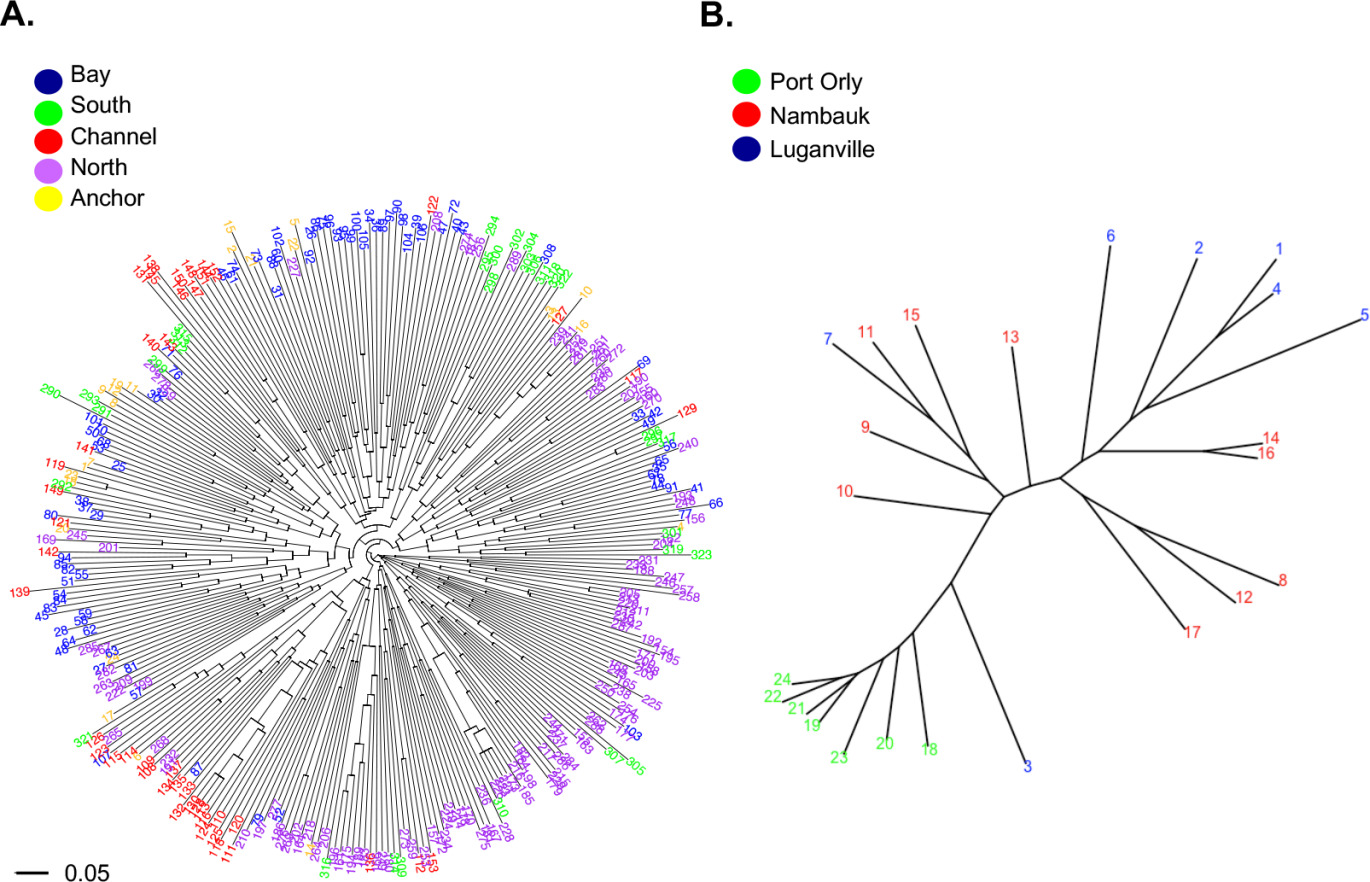
Phylogenetic analysis of *Plasmodium vivax* isolates of the Southwest Pacific. For the lower transmission regions of (A) Ngella and (B) Vanuatu, relatedness amongst haplotypes was defined by calculating the pairwise distance and visualized by drawing unrooted phylogenetic trees using the APE package in R software. Colours indicate the geographic origin of each sample as indicated in the key.

High levels of recombination in *Plasmodium* lead to large star-shaped phylogenetic trees, however genetically differentiated clades (populations) can be observed with short internal and long external branches, and when isolates are relatively closely related, structure can also be observed. Phylogenetic analysis can thus be used for recombining organisms to detect clusters of parasites that may result from local population structure or focal transmission. Phylogenetic trees support the spatial structuring of haplotypes in Solomon Islands (Ngella) and in Vanuatu (Fig 5). The tree for PNG shows no spatial clustering and thus is not shown. In Ngella, the North Coast and Bay haplotypes radiate from distinct internal branches of the tree. Two distinct clusters were also observed for Channel isolates, one of which contains a number of closely related haplotypes and falls within a clade of North Coast isolates, the other with Bay isolates (Fig 5A). In Vanuatu, haplotypes clustered by village of origin (Fig 5B).

### Changes in *Plasmodium vivax* population structure during sustained control

The availability of samples from two time points during intensive malaria control in the Solomon Islands population (Tetere 2004 and 2013) allowed the investigation of changes in *P*. *vivax* population genetic parameters in association with antimalarial interventions. In Tetere 2004, genetic diversity was high (mean *H*_s_ = 0.84, Table 1) and there was no significant multilocus LD [35] (Table 2). By 2013 however, diversity was lower with borderline significance (*H*_s_ = 0.79, p = 0.055, Wilcoxon signed rank test), the proportion of closely related haplotypes (*P*s>0.50) more than doubled, effective population sizes halved (Table 1) and multilocus LD increased to significance (Table 2). There were also low but significant levels of genetic differentiation between the two years (Jost’s D = 19.7%, Fig 2B, *F*_ST_ = 0.029, S2 Table). This suggests significant changes in the population structure of *P*. *vivax* in Tetere due to intensified malaria control.

## Discussion

As malaria-endemic countries move towards elimination of the disease, measuring changing transmission dynamics will inform control programs when to switch from broad ranging to targeted control efforts [28, 69]. Classical epidemiological studies that define the prevalence of infection are valuable as a monitoring tool, but only population genetic analyses such as those described here can detect the perturbation of transmission patterns, as indicated by the presence of inbreeding and fragmented population structure [28]. Moreover, understanding the geographic distribution and connectivity of malaria parasite populations will help to prioritize specific geographic regions for elimination [23]. Tracking the impact of control on *P*. *vivax* populations may be challenging given its more stable transmission, allowing populations to maintain high levels of diversity and gene flow relative to *P*. *falciparum* [5, 30, 70, 71]. Using the largest and most densely sampled dataset of *P*. *vivax* microsatellite genotypes to date, across a geographic region with a strong, natural gradient of transmission intensities, our results reveal a modest decrease in diversity and limited changes in the proportions of closely related haplotypes, but significant increases in multilocus LD and population structure with declining transmission. Changes in population structure were also observed between two time points in the Solomon Islands population of Tetere, revealing a similar pattern due to declining transmission with intensifying malaria control during the intervening period. Together the results suggest that sustained control efforts are needed to reduce *P*. *vivax* transmission to the point where diversity and gene flow are interrupted. This provides one possible explanation for *P*. *vivax* resilience to control and a strong incentive to maintain intensive control efforts for *P*. *vivax* for longer periods of time relative to *P*. *falciparum*.

Even with the wide range of transmission intensities investigated, the within population genetic diversity and relatedness values observed for PNG and Solomon Islands populations were similar. In Vanuatu, where *P*. *vivax* transmission has been sustained at low levels for many years, and after intensive control efforts in Tetere, Solomon Islands, lower levels of diversity and higher proportions of closely related haplotypes were observed. However, associations with multilocus LD and sub-population structure were consistently detected with declining transmission either over space or time. High *P*. *vivax* genetic diversity at low transmission was first recognized in Sri Lanka [25] and has also been observed together with significant multilocus LD in Peru [26], Malaysia [33], Indonesia [5] and Vietnam [72]. Multilocus LD and local population structure may therefore be more sensitive signals to detect changes in *P*. *vivax* transmission than diversity or relatedness. The relationship of these population genetic parameters with the proportion of polyclonal infections, suggests that polyclonality may be used as a proxy for these analyses. However, relatively small numbers of samples (n = 30–50) would need to be genotyped for the more informative population genetic analysis providing a relatively cost-effective approach to understand transmission dynamics as well as to discern connectivity between parasite populations.

The presence of identical and closely related haplotypes and significant multilocus LD in the context of high diversity is consistent with focal inbreeding which occurs as a result of low and increasingly clustered transmission. In most endemic regions, identical *P*. *vivax* haplotypes are rare and have only been seen only at very low transmission in Central Asia where the *P*. *vivax* population is nearly clonal, or at low transmission in the Amazon [19, 73]. With sustained low transmission, opportunities for recombination between diverse strains will be reduced, resulting in multilocus LD and population structure. The patterns we have observed in lower transmission areas of Solomon Islands and Vanuatu may also reflect the contribution of relapse and increasingly related clones within polyclonal infections over a sustained period of low transmission [30, 74]. Even in the high transmission setting of PNG, relapse has been shown to account for up to 80% of *P*. *vivax* infections [75] and would be expected to be even higher in a low transmission area. For some time after a reduction in transmission, the re-activation of parasites from a pool of genetically diverse parasites in the liver from numerous past infections will continue to provide opportunities for the exchange and dissemination of diverse alleles, sustaining genetic diversity in the population. As the liver reservoir is depleted over time, focal clusters of infection may be composed of more recent infections and subsequent relapses with highly related parasites [76]. Therefore, relapse is likely to maintain diverse meta-populations with high evolutionary potential. Other biological characteristics of *P*. *vivax* that are likely to sustain transmission and resilience to intervention include the pre-symptomatic and continuous production of transmission forms, coupled with efficient transmissibility at lower infection density that drives high rates of human-to-vector transmission [77, 78]. In addition, the rapid acquisition of clinical immunity early in life and low density of infection [13] would lead to a larger population reservoir of asymptomatic carriers that would not be treated [2, 7, 28]. However, unlike relapse, these features of *P*. *vivax* biology do not fully explain the patterns of population structure that we have observed in the context of declining transmission.

Across the Southwest Pacific, measures of genetic differentiation and clustering patterns using Bayesian analysis demonstrated that the diversity amongst *P*. *vivax* populations was predominantly partitioned by country of origin, which reflects both restricted gene flow and high LD in Solomon Islands and Vanuatu. One caveat is the 7–10 years gap between collections in PNG (2003–6) and the other two countries (2012–13, not including Tetere 2004), which may lead to the overestimation of population structure. However, low population structure between the 2003 PNG and 2004 Solomon Islands data was previously reported [30, 35]. In addition, comparison of PNG data from 2003 and 2005/6 revealed no evidence of genetic differentiation [30], suggesting that there are negligible changes in population structure across periods of high transmission. Population structure between the combined 2003–6 PNG data and the 2012–13 Solomon Islands data may therefore be attributed to multiple factors including the different time points, the much larger sample size from multiple Solomon Islands locations, and the intervening intensification of antimalarial interventions in Solomon Islands. The latter possibility is supported by the comparison of two time points for Tetere that reveal a decline in polyclonal infections, lower diversity and effective population size and an increase in closely related haplotypes and multilocus LD, which are the same changes that occurred with declining transmission over geographical space. Still, we cannot be certain that the population structure observed in the Solomon Islands (and Vanuatu) is the result of control efforts, because temporal population genetic data were only available for one site (Tetere).

The genetic structure of malaria parasite populations has previously been investigated with *P*. *vivax* populations over large spatial scales (e.g. between countries or distant locations within countries) [30, 32, 35, 73, 79, 80]. Local population structure was also observed with the high-resolution analyses of *P*. *vivax* population structure in the central zone of Solomon Islands, a region spanning the three island provinces of Guadalcanal (Tetere), Ngella and Malaita (Auki), an area of around 100 km^2^. Ngella *P*. *vivax* populations were also found to have moderate levels of genetic differentiation from populations of the other island provinces. Ngella is connected via a direct and popular shipping route that exists between Guadalcanal (Tetere) and Malaita (Auki) Provinces. This suggests that despite a significant level of human movement among these three provinces, importation of *P*. *vivax* cases into Ngella may be sufficiently reduced, contributing to the observed population structure. We also observed local population structure within Vanuatu. The small number of samples from each Vanuatu village limits the analysis of population structure somewhat, however, the high LD and the spatial clustering observed in the Bayesian and phylogenetic analyses would be unlikely if population structure was not present. Another caveat is that Vanuatu is at the edge of the species range, so it cannot be assumed that the low diversity and highly fragmented population structure is solely due to sustained low transmission. Indeed, fewer immigrants would be expected for a population at the edge of a species distribution, thus it is not surprising that the gene pool is smaller in this region.

On an even finer scale, within Ngella, dense sampling was done allowing resolution of population structure amongst different ecological zones and villages within each zone. *P*. *falciparum* has almost disappeared due to ongoing control interventions, but *P*. *vivax* transmission remains at a cross-sectional prevalence of around 13% by PCR [7]. Ngella *P*. *vivax* parasite populations were spatially structured among different zones and even villages within the same region. Parasite populations within Ngella (20–50km) were subdivided into four genetic clusters: Anchor/Bay/South, North Coast, and the two Channel villages. The Channel area has comparable prevalence and proportions of polyclonal infections to other Ngella areas, however the villages lay in an extensive mangrove system on both sides of a channel, suggesting that the relative isolation of these villages influences population structure. Population structure was also observed among neighbouring villages of the North Coast. Thus, *P*. *vivax* in Ngella consists of a metapopulation of several partially fragmented sub-populations [81]. No earlier samples were available from Ngella, however evidence from malaria surveys indicate a 90% reduction in cases from 1992 to 2013 (Solomon Islands National Vector Borne Diseases Control Program), consistent with the structure observed being influenced by malaria control. Sustained interventions may have led to the relatively inbred and fragmented parasite populations observed, and indicate that a critical turning point may be within reach.

Overall the results demonstrate changing *P*. *vivax* population structure with declining transmission across a gradient of high to low transmission, and to a limited extent, over time in concert with intensifying control efforts. Comparison to other populations to inform regional malaria elimination is now the subject of an ongoing study by the authors and collaborators. This systematic survey demonstrates the utility of multilocus LD and population structure to monitor *P*. *vivax* transmission. While the data indicates that transmission is high in the PNG populations, inbreeding and population substructure was observed at all spatial scales within Solomon Islands and Vanuatu, consistent with increasing recombination of related clones within populations and hampered gene flow between populations. The fact that these patterns are observed after documented transmission decline and that temporal observations in one area suggest that long term malaria control has led to these patterns, however further investigations with later time points are needed to confirm this. We conclude that while *P*. *vivax* may be more resistant to control efforts than *P*. *falciparum* [10–13], long-term sustained malaria control will reduce transmission to low levels and lead to inbreeding and fragmentation of parasite subpopulations. The results emphasize the need for interventions aiming to eliminate *P*. *vivax* to be sustained for very long periods, well beyond the time frame required for *P*. *falciparum*. Given the proposal to eliminate malaria from the Asia-Pacific by 2030 [82], intensive control pressure must be maintained to capitalize on these successes and avoid rebound. Enhanced control efforts including targeted control of fragmented populations will help to reach these goals.

## Supporting information

S1 Fig Spatial distribution of identical haplotypes across Ngella. Seven groups of identical haplotypes among 22 infections were identified. Identical haplotypes were found both within the same village (e.g. 4, 5, 7), and among villages and regions as denoted by dotted connectors (e.g. 1, 2, 3, 6).
(TIF)

S2 Fig Definition of the optimum number of clusters for the STRUCTURE analyses. The method of Evanno et al. [62] was used to calculate Delta *K* (Δ*K*) to identify the optimal of number of genetic clusters (*K*) representing the uppermost hierarchical level of population structure. The first peak represents the optimal *K* identified and this value was used in interpreting the results of each of the respective analyses A-D. Sub-population structuring may exist, which in our analyses is suggested by secondary peaks at higher K. (A) Southwest Pacific, K = 2, which was influenced by the small Vanuatu sample size (n = 24) compared to the large sample size of PNG (n = 443) and Solomon Islands (n = 420). In this instance a K of 3, was determined by considering the uneven distribution of genetic clusters amongst countries. For (B) Solomon Islands and (C) Ngella, the optimal *K* was 4. For (D) North Coast villages of Ngella, the optimal *K* was 3.
(TIF)

S1 Table Details of study sites and sample details. (DOCX)

S2 Table Estimates of multilocus linkage disequilibrium for one locus per chromosome. (DOCX)

S3 Table Alternative estimates of genetic differentiation. (DOCX)

S1 File Additional information on study sites and samples. (DOCX)

S2 File Complete dataset including all microsatellite haplotypes used in the analysis. (XLSX)
