# Supplementary figures and images for: Increasingly inbred and fragmented populations of Plasmodium vivax associated with the eastward decline in malaria transmission across the Southwest Pacific
Source: PLoS Negl Trop Dis. 2018 Jan 26;12(1):e0006146. doi: 10.1371/journal.pntd.0006146 (PMC5802943; doi:10.1371/journal.pntd.0006146)

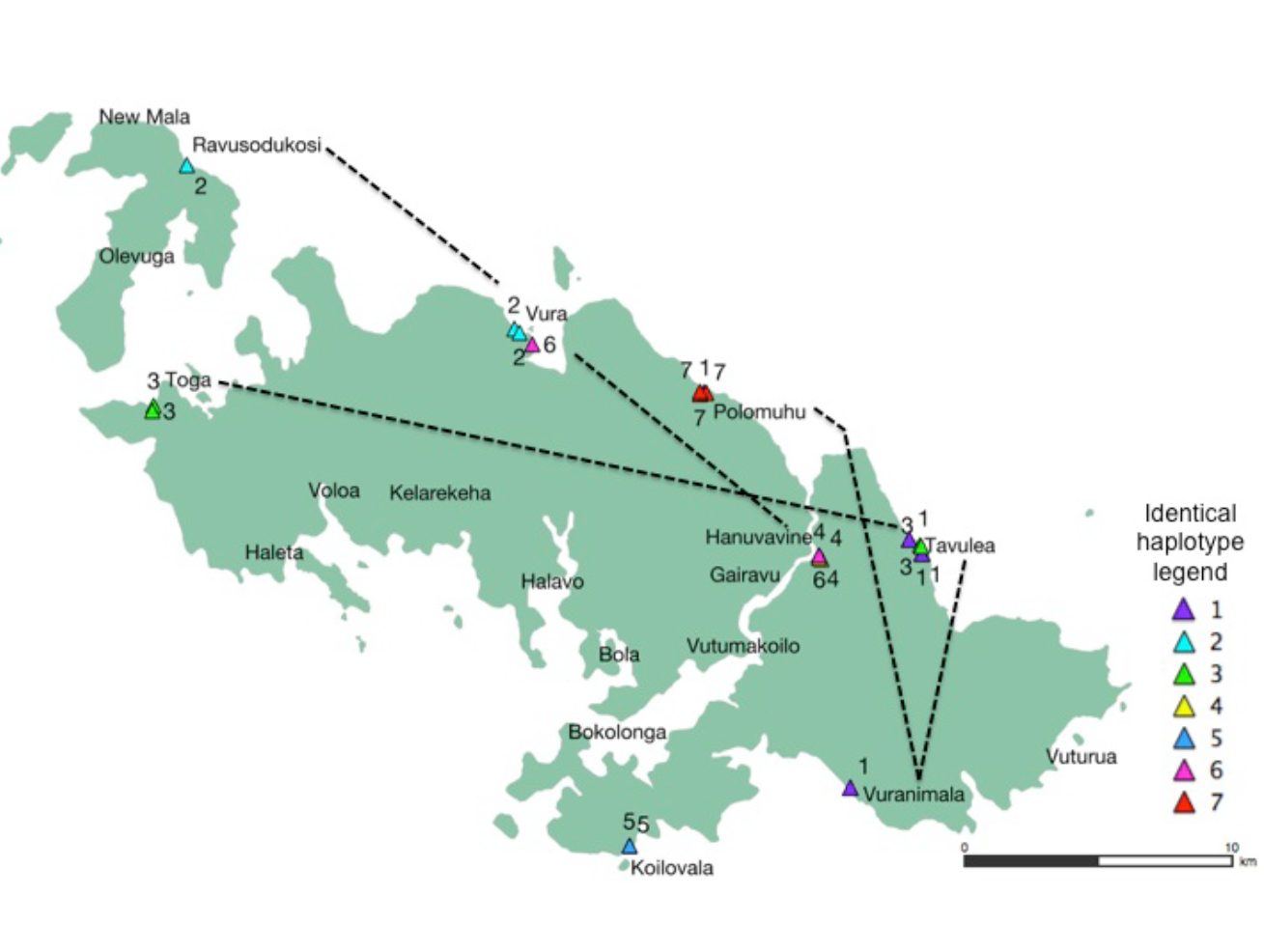

Supplement: S1 Fig — Seven groups of identical haplotypes among 22 infections were identified. Identical haplotypes were found both within the same village (e.g. 4, 5, 7), and among villages and regions as denoted by dotted connectors (e.g. 1, 2, 3, 6). (TIF) [file pntd.0006146.s001.tif]

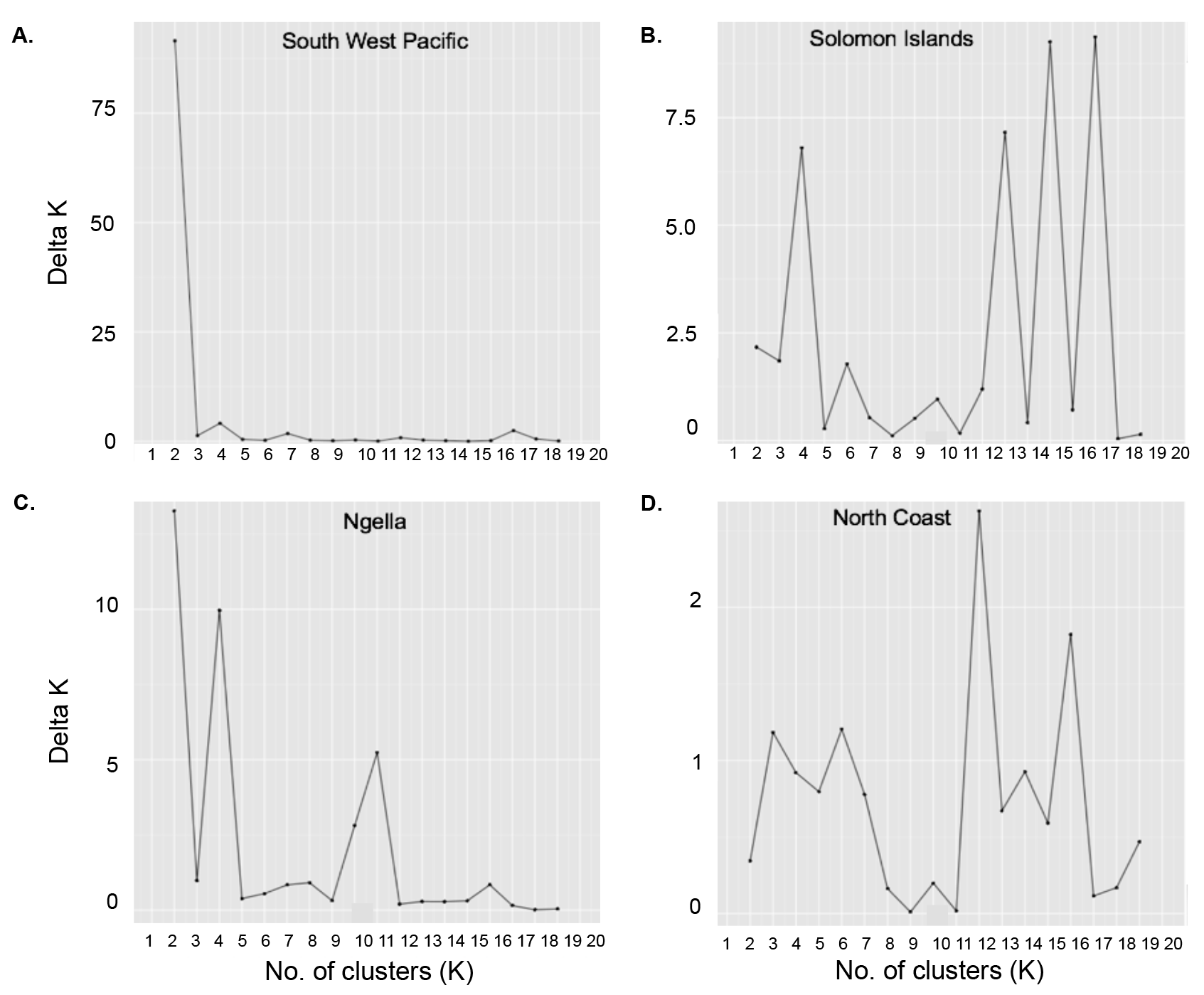

Supplement: S2 Fig — The method of Evanno et al. [62] was used to calculate Delta K (ΔK) to identify the optimal of number of genetic clusters (K) representing the uppermost hierarchical level of population structure. The first peak represents the optimal K identified and this value was used in interpreting the results of each of the respective analyses A-D. Sub-population structuring may exist, which in our analyses is suggested by secondary peaks at higher K. (A) Southwest Pacific, K = 2, which was influenced by the small Vanuatu sample size (n = 24) compared to the large sample size of PNG (n = 443) and Solomon Islands (n = 420). In this instance a K of 3, was determined by considering the uneven distribution of genetic clusters amongst countries. For (B) Solomon Islands and (C) Ngella, the optimal K was 4. For (D) North Coast villages of Ngella, the optimal K was 3. (TIF) [file pntd.0006146.s002.tif]
