# Supplementary material for: Increasingly inbred and fragmented populations of Plasmodium vivax associated with the eastward decline in malaria transmission across the Southwest Pacific
Source: PLoS Negl Trop Dis. 2018 Jan 26;12(1):e0006146. doi: 10.1371/journal.pntd.0006146 (PMC5802943; doi:10.1371/journal.pntd.0006146)
Supplement: S1 Table — (DOCX) [file pntd.0006146.s003.docx]

**S1 Table**. **Study sites and sample details.**

| **Country** | **Province** | **Catchment Area** | **Year** | **Survey type** | ***P. vivax PCR* prevalence (%)** | **No. samples genotyped for MS16/msp1F3^c^** | **Polyclonal infections (%)** | **Reference** |
| --- | --- | --- | --- | --- | --- | --- | --- | --- |
| Papua New Guinea | East Sepik | Ilaita, Kunjingini, Wosera | 2003-2007 | Cross-sectional and longitudinal | 15-53^a^ | 1350 | 74.3 | [35, 79] |
|  | Madang | Alexishafen, Malala, Mugil, Utu | 2006 | Cross-sectional | 32^b^ | 321 | 52.2 | [30, 35, 79] |
|  | Simbu | Sigimaru | 2004-2005 | Cross-sectional | n.a. | 48 | 65.7 | [35] |
| Solomon Islands | Guadalcanal | Tetere | 2004-2005 | Clinical | n.a. | 68 | 88.2 | [35] |
|  |  | Tetere | 2013 | Clinical | n.a. | 56 | 58.6 | This publication |
|  | Central | Ngella Islands (all) | 2012 | Cross-sectional | 13.4 | 373 | 28.7 | [7] |
|  |  | Bay |  | Cross-sectional | 13.7 | 105 | 21.0 | [7] |
|  |  | South |  | Cross-sectional | 9.9 | 34 | 20.6 | [7] |
|  |  | Channel |  | Cross-sectional | 13.2 | 54 | 27.8 | [7] |
|  |  | North |  | Cross-sectional | 31.7 | 160 | 36.9 | [7] |
|  |  | Anchor |  | Cross-sectional | 3.9 | 20 | 20.0 | [7] |
|  | Malaita | Auki | 2013 | Clinical | n.a. | 18 | 27.8 | This publication |
| Vanuatu | Sanma | Espiritu Santo | 2013 | Clinical | n.a. | 25 | 12.0 | This publication |

n.a. = prevalence measurements not available as these samples were collected from confirmed *P. vivax* clinical cases.

^a^Data for Ilaita and Wosera only, ^b^Data for Malala, Mugil and Utu only, ^c^Number of samples with at least one marker successfully typed.
