# Supplementary material for: Increasingly inbred and fragmented populations of Plasmodium vivax associated with the eastward decline in malaria transmission across the Southwest Pacific
Source: PLoS Negl Trop Dis. 2018 Jan 26;12(1):e0006146. doi: 10.1371/journal.pntd.0006146 (PMC5802943; doi:10.1371/journal.pntd.0006146)
Supplement: S2 Table — (DOCX) [file pntd.0006146.s004.docx]

**S2 Table**. **Estimates of Multilocus Linkage Disequilibrium in *Plasmodium vivax* populations of the Southwest Pacific including only one locus per chromosome (MS5 and MS15 excluded)**

| **Population** | **Subpopulation** | **All haplotypes, 1 locus per chromosome** | | | **Confirmed monoclonal haplotypes, 1 locus per chromosome** | | |
| --- | --- | --- | --- | --- | --- | --- | --- |
|  |  | n | *I*_A_^S^ | *p* | n | *I*_A_^S^ | *p* |
| **Tetere 2004** |  | 21 | 0.022 | 0.0976 | 0 | n.a. | n.a. |
| **Tetere 2013** |  | 31 | 0.018 | 0.0519 | 16 | 0.037 | 0.0435 |
| **Auki** |  | 9 | 0.082 | 0.025 | 6 | 0.022 | 0.423 |
| **Ngella** |  | 165 | 0.025 | <0.00001 | 61 | 0.04 | <0.00001 |
|  | Bay | 32 | 0.038 | 0.0081 | 9 | 0.087 | 0.0267 |
|  | South | 17 | 0.065 | 0.0036 | 7 | 0.13 | 0.012 |
|  | Channel | 29 | 0.076 | 0.0001 | 9 | 0.087 | 0.0249 |
|  | North | 73 | 0.041 | <0.00001 | 28 | 0.09 | <0.00001 |
|  | Anchor | 14 | 0.11 | 0.0002 | 8 | 0.019 | 0.377 |
| **Vanuatu** |  | 22 | 0.154 | <0.00001 | 10 | 0.189 | 0.00002 |
| **TOTAL** |  | 248 | n.d. | n.d. | 93 | n.d. | n.d. |

Complete and unique haplotypes only were used for the analysis, by discarding all but one of the seven clonal haplotypes identified. n = number of haplotypes used in the analysis. I_A_^S^ = Index of Association from LIAN analysis, MOI = multiplicity of infection, n.a. = not available due to sample size constraints. n.d. = not done.
