# Supplementary material for: Increasingly inbred and fragmented populations of Plasmodium vivax associated with the eastward decline in malaria transmission across the Southwest Pacific
Source: PLoS Negl Trop Dis. 2018 Jan 26;12(1):e0006146. doi: 10.1371/journal.pntd.0006146 (PMC5802943; doi:10.1371/journal.pntd.0006146)
Supplement: S3 Table — (DOCX) [file pntd.0006146.s005.docx]

**S3 Table**. **Alternative estimates of genetic differentiation**

*G*_ST_ on the lower left; and *F*_ST_ on upper right

**Southwest Pacific**

|  | PNG | Solomon Islands | Vanuatu |
| --- | --- | --- | --- |
| PNG |  | 0.20 | 0.053 |
| Solomon Islands | 0.038 |  | 0.054 |
| Vanuatu | 0.085 | 0.085 |  |

**Solomon Islands**

|  | Tetere 2004 | Tetere 2013 | Ngella | Auki |
| --- | --- | --- | --- | --- |
| Tetere 2004 |  | 0.029 | 0.046 | 0.046 |
| Tetere 2013 | 0.021 |  | 0.024 | 0.024 |
| Ngella | 0.028 | 0.015 |  | 0.040 |
| Auki | 0.039 | 0.029 | 0.034 |  |
