# Supplementary material for: Increasingly inbred and fragmented populations of Plasmodium vivax associated with the eastward decline in malaria transmission across the Southwest Pacific
Source: PLoS Negl Trop Dis. 2018 Jan 26;12(1):e0006146. doi: 10.1371/journal.pntd.0006146 (PMC5802943; doi:10.1371/journal.pntd.0006146)
Supplement: S1 File — (DOCX) [file pntd.0006146.s006.docx]

**S1 File. Additional information on study sites and samples**

**Papua New Guinea.** Previously published microsatellite haplotype data were available from three areas Madang Province (n=175), East Sepik Province (n=229) and Simbu Province (Sigimaru, n=39) [1-3], and represent both asymptomatic and clinical samples collected during cross-sectional and cohort studies in 2005-2006 prior to the implementation of widespread intensified control. Full details of these samples are available in the relevant publications.

**Solomon Islands.** Malaria transmission in the Solomon Islands is highly heterogeneous throughout the country, with the highest number of cases in Central Islands, Guadalcanal and Malaita provinces [4], the three of the most populous provinces (approximately 40% of national population [5]). In 2013, *P. vivax* accounted for 47.6% of all confirmed clinical cases of malaria nationwide and it was the predominant species circulating among asymptomatic individuals in the community [6-8].

Solomon Islands *P. vivax* haplotypes originated from isolates collected in three provinces, namely Central Province (Ngella), Guadalcanal (Tetere) and Malaita (Auki). The three provinces are separated by sea (Figure 1), but are well connected by a frequent and popular ferry service and numerous, unscheduled motorized boat trips. Ngella is a group of three islands, namely Anchor, Big Ngella and small Ngella. Tetere is located 30 km east of the national capital Honiara, on Guadalcanal Island and Auki is the capital of Malaita province.

The Ngella *P. vivax* isolates were collected as part of a large household-based, cross–sectional survey of 3501 participants of all ages conducted in May-June 2012 [8]. This survey demonstrated Ngella to be an area of considerable residual *P. vivax* (prevalence by PCR =13.4%, 468/3501), but disappearing *P. falciparum* transmission (prevalence by PCR =0.14%, 5/3501) [8].

Tetere isolates were collected at two time points during a period of intensified malaria control. Tetere 2004 isolates were collected from a clinical trial conducted between 2004-5 [9]. This microsatellite data was described elsewhere previously (n=45, [3, 9]). During that time, Tetere was considered as high transmission, and the study which collected the isolates, reported a *P. vivax* prevalence of 19.1% by light microscopy (LM) [9], a diagnostic method which is substantially less sensitive than the PCR methods used in the Ngella survey [8]. Tetere 2013 isolates were clinical samples collected in the context of an antimalarial drug efficacy trial conducted in 2013 (Lyndes Wini, personal communication).

Samples from Auki were also from a clinical trial conducted in 2013 (Wini *et al*., unpublished). Prevalence data was not available for the Tetere 2013 nor the Auki study.

**Vanuatu.** In 2013, a similar clinical trial was carried out on Espiritu Santo, with clinical samples being collected from participants residing in three villages Port Orly, Nambauk and Luganville (Sarah Boyd, personal communication). Prevalence data was not available for this area.

**REFERENCES**

1. Arnott A, Barnadas C, Senn N, Siba P, Mueller I, Reeder JC, et al. High genetic diversity of *Plasmodium vivax* on the north coast of Papua New Guinea. Am J Trop Med Hyg. 2013;89(1):188-94.

2. Jennison C, Arnott A, Tessier N, Tavul L, Koepfli C, Felger I, et al. *Plasmodium vivax* populations are more genetically diverse and less structured than sympatric *Plasmodium falciparum* populations. PLoS neglected tropical diseases. 2015;9(4):e0003634.

3. Koepfli C, Timinao L, Antao T, Barry AE, Siba P, Mueller I, et al. A Large *Plasmodium vivax* Reservoir and Little Population Structure in the South Pacific. PloS one. 2013;8(6):e66041.

4. Pacific Malaria Initiative Support Centre (PacMISC). Malaria on isolated Melanesian islands prior to the initiation of malaria elimination activities. Malaria journal. 2010;9:218.

5. Solomon Islands Government. Volume I Report on 2009 Population And Housing

Census: Basic Tables and Census Description. Honiara, Solomon Islands: 2009.

6. Harris I, Sharrock WW, Bain LM, Gray KA, Bobogare A, Boaz L, et al. A large proportion of asymptomatic Plasmodium infections with low and sub-microscopic parasite densities in the low transmission setting of Temotu Province, Solomon Islands: challenges for malaria diagnostics in an elimination setting. Malaria journal. 2010;9:254.

7. National Vector Borne Diseases Control Program of the Solomon Islands. Annual Malaria Report 2012. Honiara, Solomon Islands: Ministry of Health, 2013.

8. Waltmann A, Darcy AW, Harris I, Koepfli C, Lodo J, Vahi V, et al. High Rates of Asymptomatic, Sub-microscopic *Plasmodium vivax* Infection and Disappearing *Plasmodium falciparum* Malaria in an Area of Low Transmission in Solomon Islands. PLoS neglected tropical diseases. 2015;9(5):e0003758.

9. Ballif M, Hii J, Marfurt J, Crameri A, Fafale A, Felger I, et al. Monitoring of malaria parasite resistance to chloroquine and sulphadoxine-pyrimethamine in the Solomon Islands by DNA microarray technology. Malaria journal. 2010;9:270.
